# Supplementary material for: The Joint Evolution of Herbivory Defense and Mating System in Plants: A Simulation Approach
Source: Plants (Basel). 2023 Jan 26;12(3):555. doi: 10.3390/plants12030555 (PMC9919119; doi:10.3390/plants12030555)
Supplement: Supplementary file 1 [file plants-12-00555-s001.zip › plants-1983177-supplementary.pdf]

Supplementary Information for:

The joint evolution of resistance and tolerance to herbivory and the mating system in plants: A simulation approach

Edson Sandoval-Castellanos[1], Juan Núñez-Farfán[1,*]

[1]Laboratorio de Genética Ecológica y Evolución, Departamento de Ecología Evolutiva, Instituto de Ecología, Universidad Nacional Autónoma de México, *Apartado Postal 70-275, Código Postal 04510, Distrito Federal, México*

*Contact information: farfan@unam.mx

Contents:

1. Supplementary Text S1. Estimation of the Number of Lethal Recessive Alleles
2. Supplementary Figure S1. Summary statistics of simulations: resistance and tolerance costs.
3. Supplementary Figure S2. Summary statistics of simulations: inbreeding depression.
4. Supplementary Figure S3. Summary statistics of simulations: linearity and inbreeding depression.
5. Supplementary Text S2. Algorithm for Simulation of Plants and Herbivores Populations.
6. Supplementary Text S3. Special cases.
7. Supplementary Figure S4. Fitness surface as function of resistance and tolerance.
8. Supplementary Figure S5. Special cases.
9. Supplementary Figure S6. Evolution of selfing for various initial ratios.

Supplementary Text S1

Estimation of the Number of Lethal Recessive Alleles

Calculating the expected number of lethal mutations that a single (plant) genome requires to reach equilibrium is the same as calculating the intra-genomic frequency of lethal mutations that produces an expected number of (lethal) homozygotes equal to the mutation rate. Let the lethal mutation rate be u and its intra-genomic frequency at generation t be q_t. The expected number of intragenomic lethal homozygous genes (h_{om}) depends on the number of genes in the genome (g_s), in a similar way than the Hardy-Weinberg law: $h_{om} = g_s \times q^2$. The frequency of lethals in a genome at a given time q_{t+1} can be obtained in terms of the frequency of a previous generation q_t in a genome (assuming that in average genomes, each generation would tend to have the same lethal frequencies). If $p_{t-1} = 1-q_{t-1}$ and $v = u/2g_s$ (increment of lethal frequency obtained via mutation), then $q_{t+1} = \frac{(p_t - v)(q_t + v)}{1-(q_t + v)^2}$ …(a) for outcrossing genomes. For selfing genomes, a reduction of heterozygosis by 50% each generation must be taken into account, yielding $q_{t+1} = \frac{(p_t - v)(q_t + v)}{2\left(1-(p_t - v)(q_t + v)-(q_t + v)^2\right)}$ …(b). Thus, equilibrium for outcrossing and selfing genomes occurs when $q_{t+1} = q_t$, leading equations (a) and (b) to become third- and second-degree equations, respectively. From all the solutions those equations have, the ones in the interval [0, 1] are the ones corresponding to equilibrium frequencies of lethal alleles. Because the expressions for those solutions are too long, only the equations are presented (solutions can be obtained easily with calculators available online): $q^3+q^2(2v-1)+q(v^2-2v)+v-v^2 = 0$ for outcrossing genomes and $q^2-q+v-v^2 = 0$ for selfing genomes.

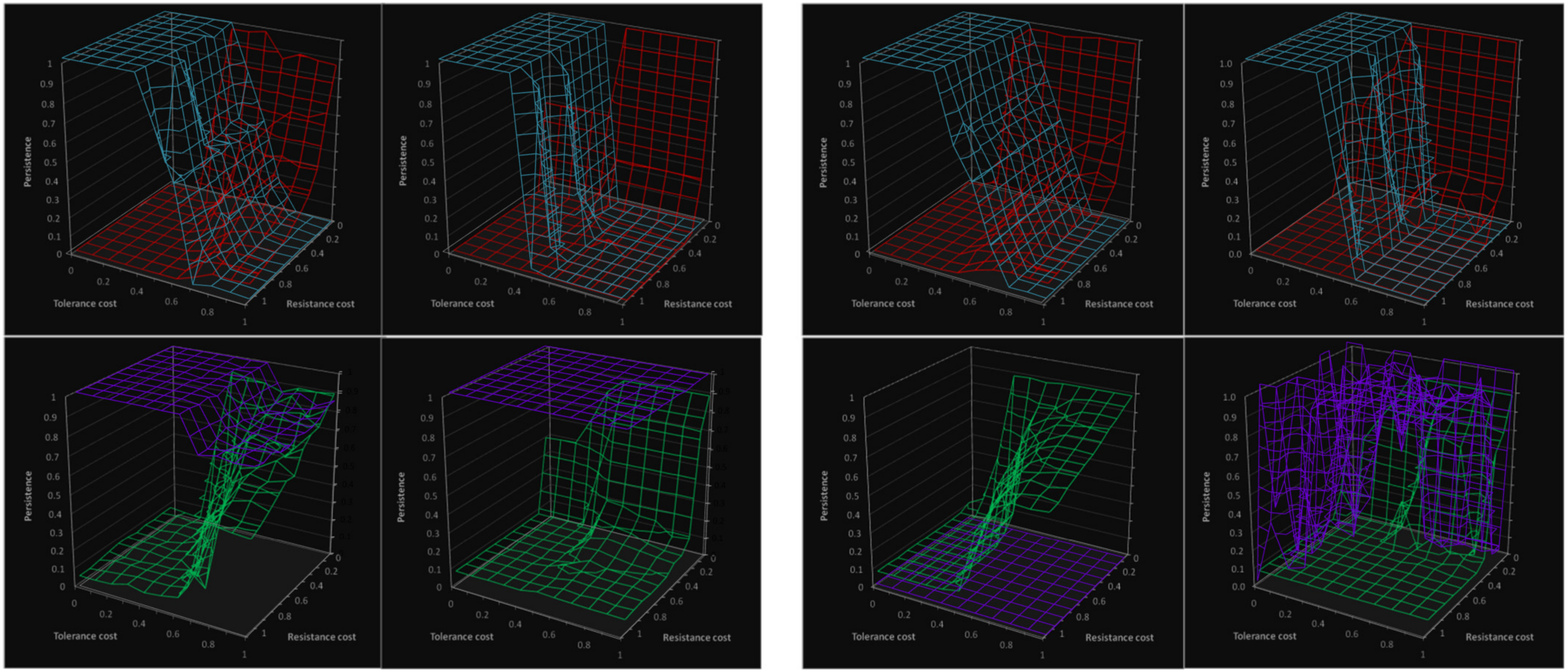

Multiplicative Additive Multiplicative Additive

Heritable selfing Non-Heritable selfing

Supplementary Figure S1. Summary statistics of simulations: resistance and tolerance costs. Upper panels show the persistence of plant resistance (red) and tolerance (blue) as functions of different combinations of resistance and tolerance costs. Tolerance benefit (b_t) also was set inversely proportional to the tolerance cost: $b_t = 1\text{-}c_t$, for instance if $c_t = 0.3$ then $b_t = 0.7$. Lower panels show the persistence of herbivore anti-resistance (green), and plant selfing (purple) also as functions of the resistance and tolerance costs in plants.

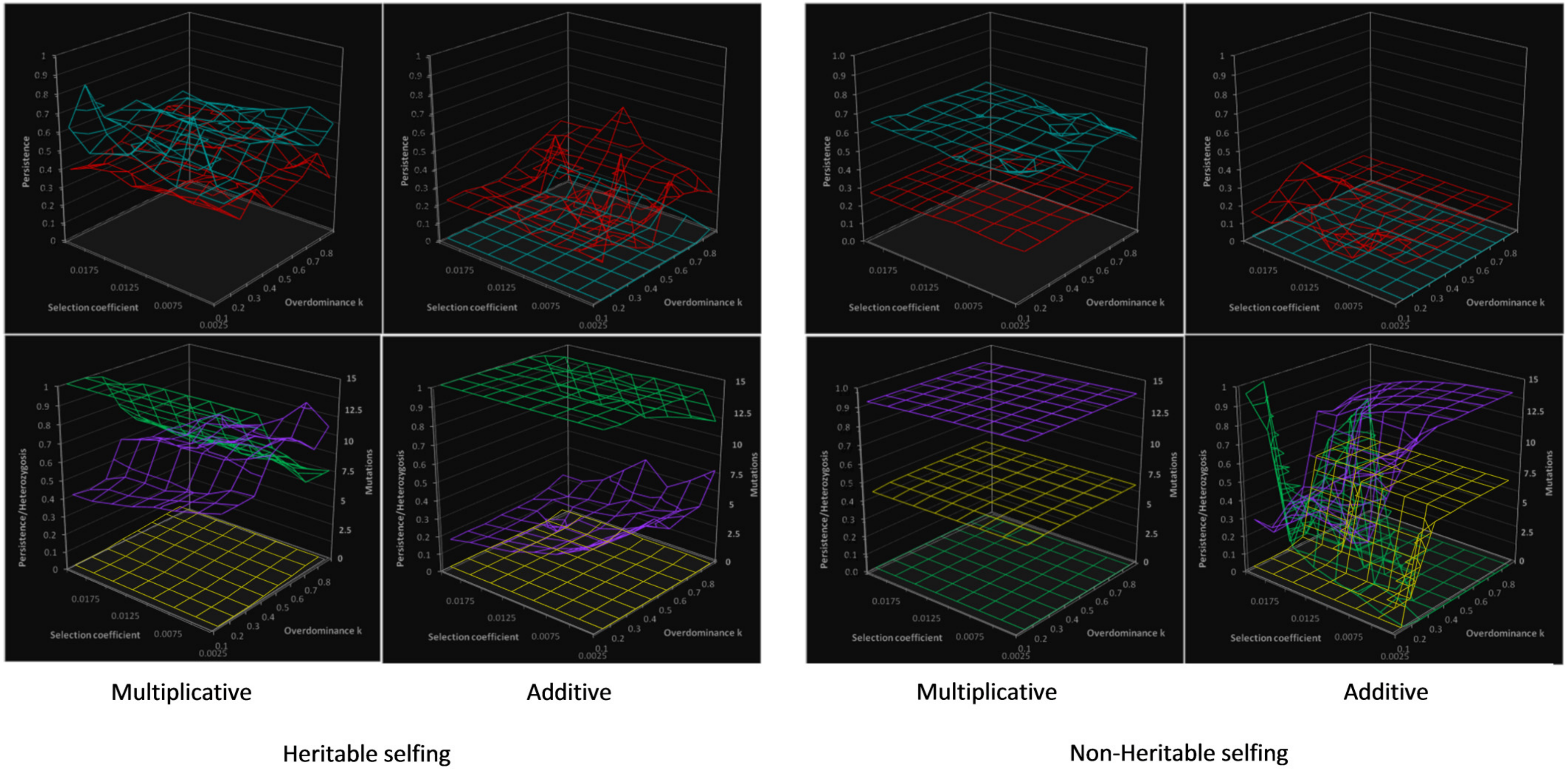

Supplementary Figure S2. Summary statistics of simulations: inbreeding depression. Summary statistics surfaces are displayed as functions of two ID components: dominance intensity given by the selection coefficient of harmful alleles (*s*); and overdominance intensity given by the parameter *k*. Upper panels show the persistence of plant resistance (red) and tolerance (blue) as functions of ID components. Lower panels show plant selfing persistence (green), average number of harmful alleles per plant over generations (purple), and average individual genomic heterozygosis over generations (yellow) as functions of ID components.

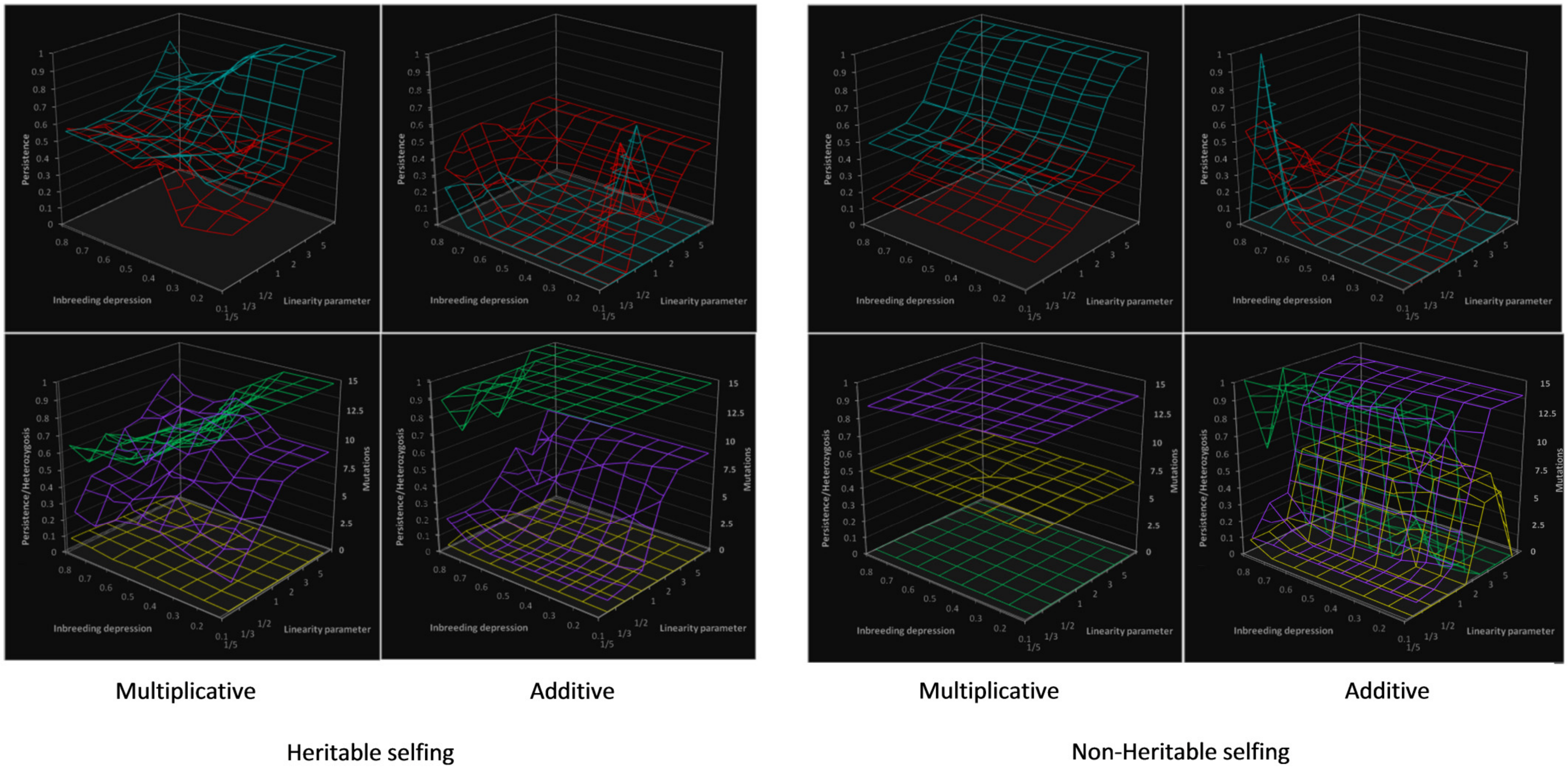

Supplementary Figure S3. Summary statistics of simulations: linearity and inbreeding depression. The summary statistics here are displayed as functions of two variables: 1) the linearity parameter; and 2) the inbreeding depression intensity (given by increasing jointly the components of dominance and overdominance –see caption to supplementary figure 2 for its definition-). Upper graphics show the persistence of plant resistance (red) and tolerance (blue) in the simulated populations for different combinations of *l* and ID intensity. Lower panels show plant selfing persistence (green), average number of harmful alleles per plant over generations (purple), and average individual genomic heterozygosis over generations (yellow). Scale of inbreeding depression only shows *k* values but they also have associated *s* values as follows: $s = 0.0025/k = 0.1$; $s = 0.005/k = 0.2$; $s = 0.0075/k = 0.3$; $s = 0.01/k = 0.4$; $s = 0.0125/k = 0.5$; $s = 0.015/k = 0.6$; $s = 0.0175/k = 0.7$; $s = 0.02/k = 0.8$.

Supplementary Text S2

Algorithm for Simulation of Plants and Herbivores Populations.

Each individual plant and herbivore got assigned an array with the information regarding its sex (for herbivores), genotype (for resistance, tolerance and selfing genes in plants; for anti-herbivore genes in herbivores), genomic heterozygosis and number of accumulated harmful alleles. The generational cycles consisted of the following stages:

The herbivory and herbivores reproduction was programmed as follows: random plant and herbivore genotypes were compared according with a gene-for-gene model. If the herbivore had the right genotype, herbivory was considered successful and the herbivore was selected for reproduction. In addition, herbivores' anti-resistance genes mutated to the alternative allele at a probability of 0.01 per generation. The number of descendants of each reproduction event had a Poisson probability distribution ($\lambda = 25$). The obtained value was then adjusted by the cost of anti-resistance (see Table 1). Reproduction stopped when the next generation was complete or the set of parents was used up.

The plants reproduction, unlike the herbivores, involved one main parent (who produced the seeds) and several pollen donors. The number of descendants was drawn also from a Poisson distribution ($\lambda = 20$) and adjusted with Equation (1) or (2). The number of harmful mutations was calculated from a binomial deviate with a sample size of $2g$ (g := genome size; set to 100 genes) and $p = u_t/4g$ (u_t := number of harmful mutations in both parents). Harmful mutations were lethal when homozygous. The individual heterozygosis of the descendants was calculated in a way that was analogue to the Hardy-Weinberg law, resulting in a reduction in the selfing offspring of approximately 50% per generation (see Sup. Appendix S1). Reproduction stopped when the next generation was complete or the set of parents got used up. Also, the plant population received a migrant with a random genotype each generation.

Supplementary Text S3

Special Cases.

A. **Rise and fall of selfing.** The pattern depicted in Supplementary Figure S4A is the result of the *rcs*. It has three stages: *i*) in the initial stage, both herbivores' anti-resistance and plants' resistance frequencies are high, but they decrease due to costs. During this stage, the selfing plants lose heterozygosis and also their resistance alleles; *ii*) antagonistic waves of selfing and resistance are established by the *rcs* and also because the selfing plants have lost resistance already. The waving pattern is originally established by the frequency-dependent selection dynamic between plants' resistance and herbivores' anti-resistance, and it is enhanced by *rcs*; *iii*) a large incursion of selfing, which began via drift, takes selfing almost to fixation, while it depletes resistance. Then, if ID is low ($k < 0.3$, $s < 0.0125$), selfing reaches fixation, and the population remains without change; however, if ID is strong, the joint effect of ID and the *rcs* exerts extra pressure on selfing, which slows down before finally collapsing. At this point, *rcs* is effective because resistance has become advantageous again due to the reduction of herbivores' anti-resistance. Afterwards, the waving dynamic is reestablished. This pattern only occurs under the additive model with non-heritable selfing and moderate ID. Interestingly, tolerance always became fixed, so a mixed strategy and a mixed mating system were present most of the time.

B. **Resistance-tolerance incursions via migration.** Some simulations displayed isolated waves for which resistance and tolerance had an extremely high positive or negative correlation (Supplementary Figure S4B). Those waves appeared in populations in which resistance and tolerance had been previously lost or fixed and non-heritable selfing was also fixed. The positively correlated waves corresponded to migrants containing both resistance and tolerance that invaded the population, given their inability to mate with selfers and a transient advantage of resistance and/or tolerance. Then, the rise of anti-

resistance in herbivores and the automatic advantage of selfers caused the decline of the wave.

C. **Anti-resistance multiple equilibriums.** In some simulations, the herbivores' anti-resistance shifted among several plateaus, in spite of having similar levels of plant resistance. The cause of the intermediate levels was the fixation in some, but not all, of the several anti-resistance genes, while the higher and lower plateaus were determined by the mutation rate (0.01 per allele per generation), which prevented complete loss or fixation (Supplementary Figure S4C).

D. **Good waves, bad waves.** Resistance made high-amplitude well-shaped waves privative of fixed selfing, while at other times, it made short, noisy waves that occurred both with selfing and outcrossing (Supplementary Figure S4D). This difference was due to the fixation of alleles and the loss of resistance in selfing organisms; the amplitude of the waves reflected the intensity of the trade-off between resistance and selfing.

E. **Flat persistence.** The presence of perfectly stationary resistance or tolerance in time at a frequency of 0.5 (Supplementary Figure S4E) occurred because of the fixation of the effect allele at one gene and the neutral allele at another gene (recall that plants always had two genes for both resistance and tolerance). This behavior was promoted by two conditions: fixed selfing (fixed homozygosis) in plants, and resistance and tolerance costs that made the fitness of the double homozygote plants (in repulsion) similar to that of the alternative genotypes. Such patterns reflected a tight connection between selfing, resistance, and tolerance. For example, for parameter values of $c_r = 0.0$, $c_t = 0.0$, and $b_t = 1.0$, the pattern occurred around 30% of the time, revealing that double homozygosis (in repulsion) did not have a higher fitness than other genotypes.

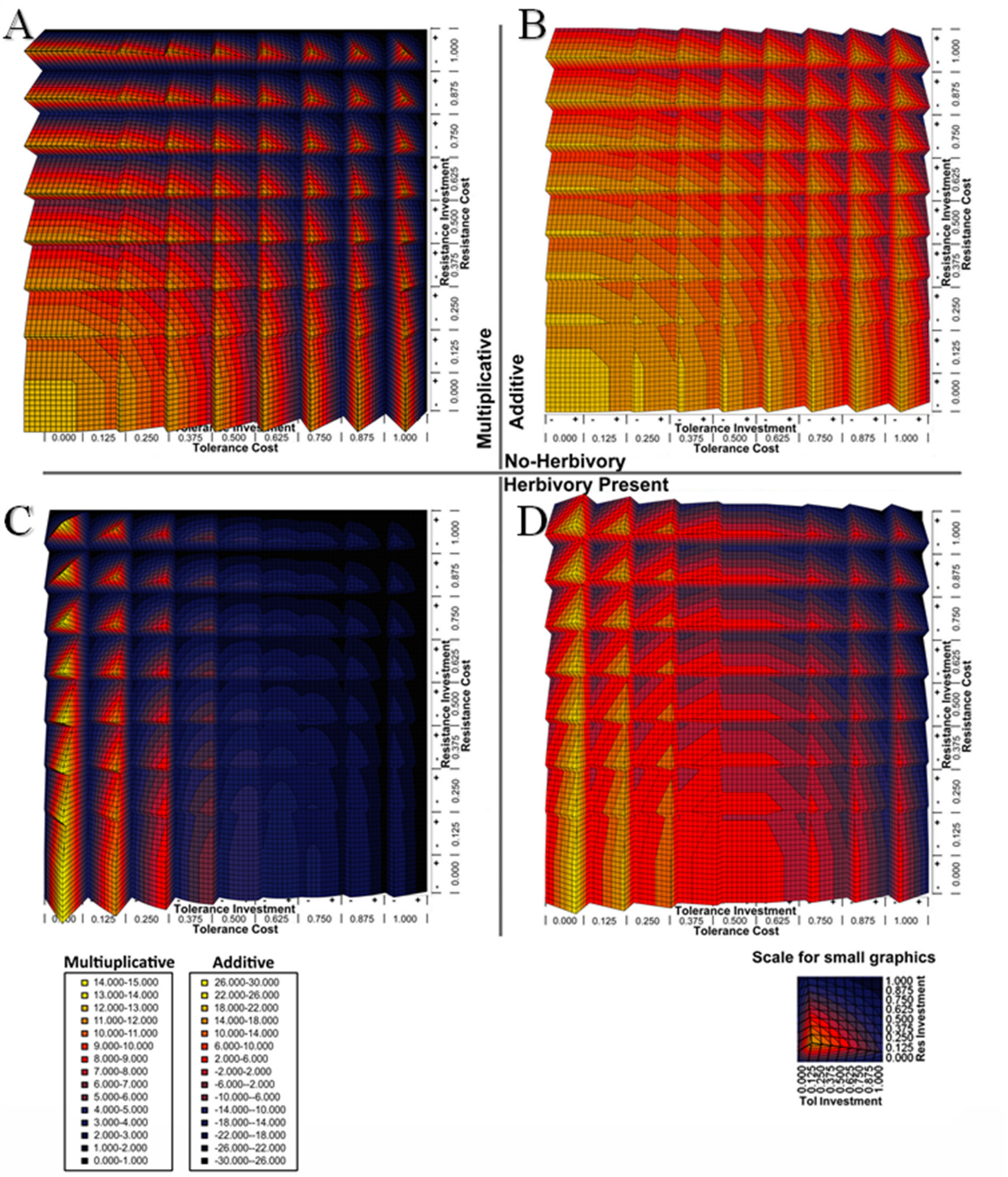

Supplementary Figure S4. Fitness surface as function of resistance and tolerance. Charts show the fitness as the equivalent of a 5-D graph (projected in 2-D) in which fitness is a function of four variables: costs and investments of both resistance and tolerance. The large chart can be seen as the composite of many small graphics where the fitness surface is a function of resistance and tolerance investments whereas the large scale changes in terms of resistance and tolerance costs. The tolerance benefit was set to 1-c_t. Panels A and C were obtained under the multiplicative model while panels B and D under the additive model, and panels A and B correspond to non-herbivory while panels C and D are the cases in which herbivory was present (damage was set to 75%). Recall that resistance benefits depended on complex interactions with herbivores and are not represented here.

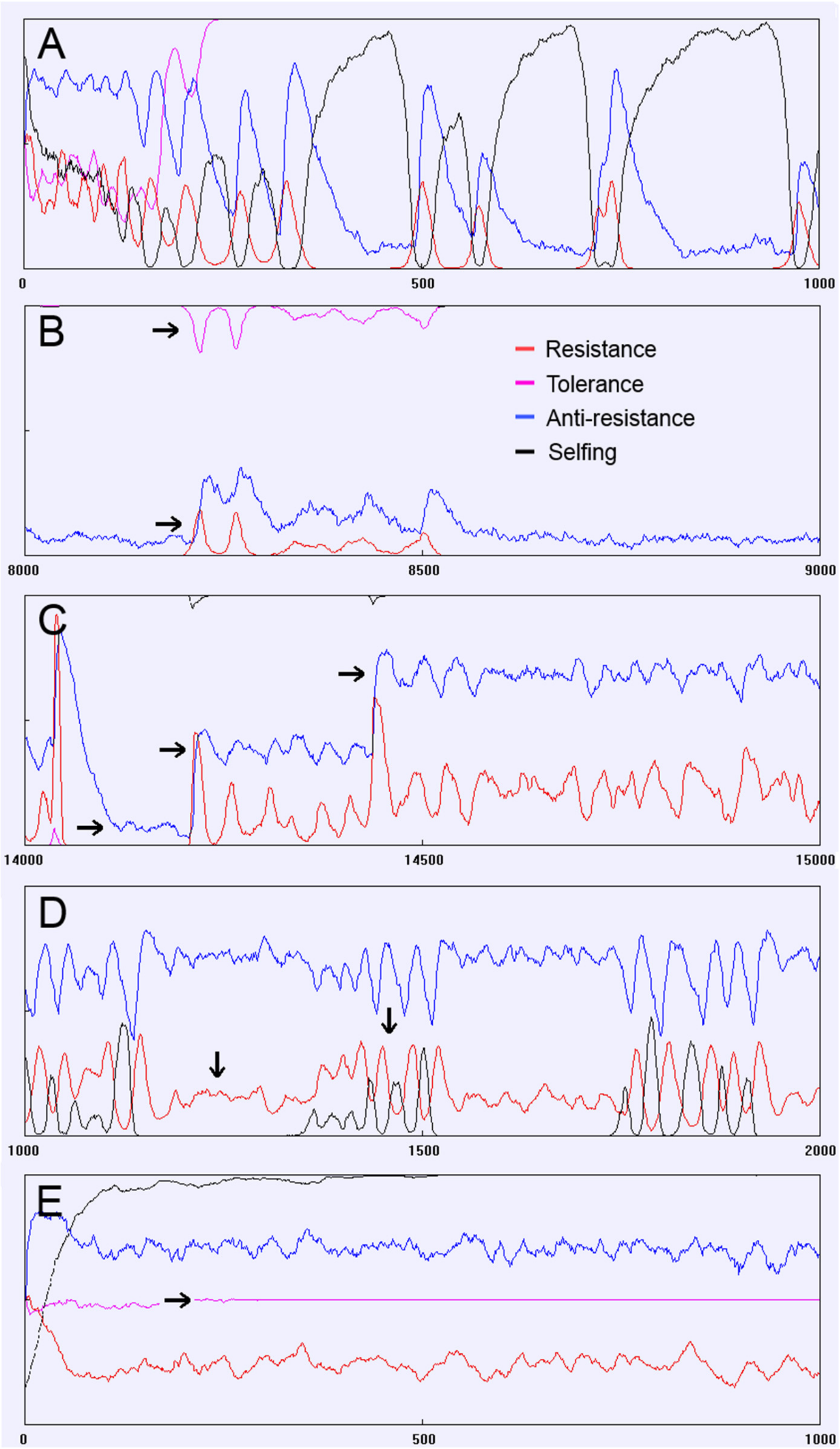

Supplementary Figure S5. Special cases. Graphics showing the evolution of (allele) frequencies of resistance, tolerance, selfing, and herbivores anti-resistance over generations, obtained on specific simulation runs in order to show the intriguing patterns analyzed as special cases (see text). Parameter and settings used were, A) $c_r = 0.1$, $c_t = 0.5$, $b_t = 0.5$, $k = 0.3$, $H = 0.25$, $s = 0.005$, $l = 1.0$, additive model, and non-heritable selfing; B) $c_r = 0.15$, $c_t = 0.4$, $b_t = 0.25$, $k = 0.2$, $H = 0.25$, $s = 0.02$, $l = 1.0$, additive model, and non-heritable selfing; C) $c_r = 0.15$, $c_t = 0.4$, $b_t = 0.25$, $k = 0.1$, $H = 0.25$, $s = 0.0175$, $l = 1.0$, additive model, and non-heritable selfing; D) $c_r = 0.15$, $c_t = 0.4$, $b_t = 0.25$, $k = 0.5$, $H = 0.25$, $s = 0.0075$, $l = 1.0$, additive model, and heritable selfing; E) $c_r = 0.1$, $c_t = 0.5$, $b_t = 0.5$, $k = 0.3$, $H = 0.25$, $s = 0.005$, $l = 1.0$, multiplicative model, and heritable selfing.

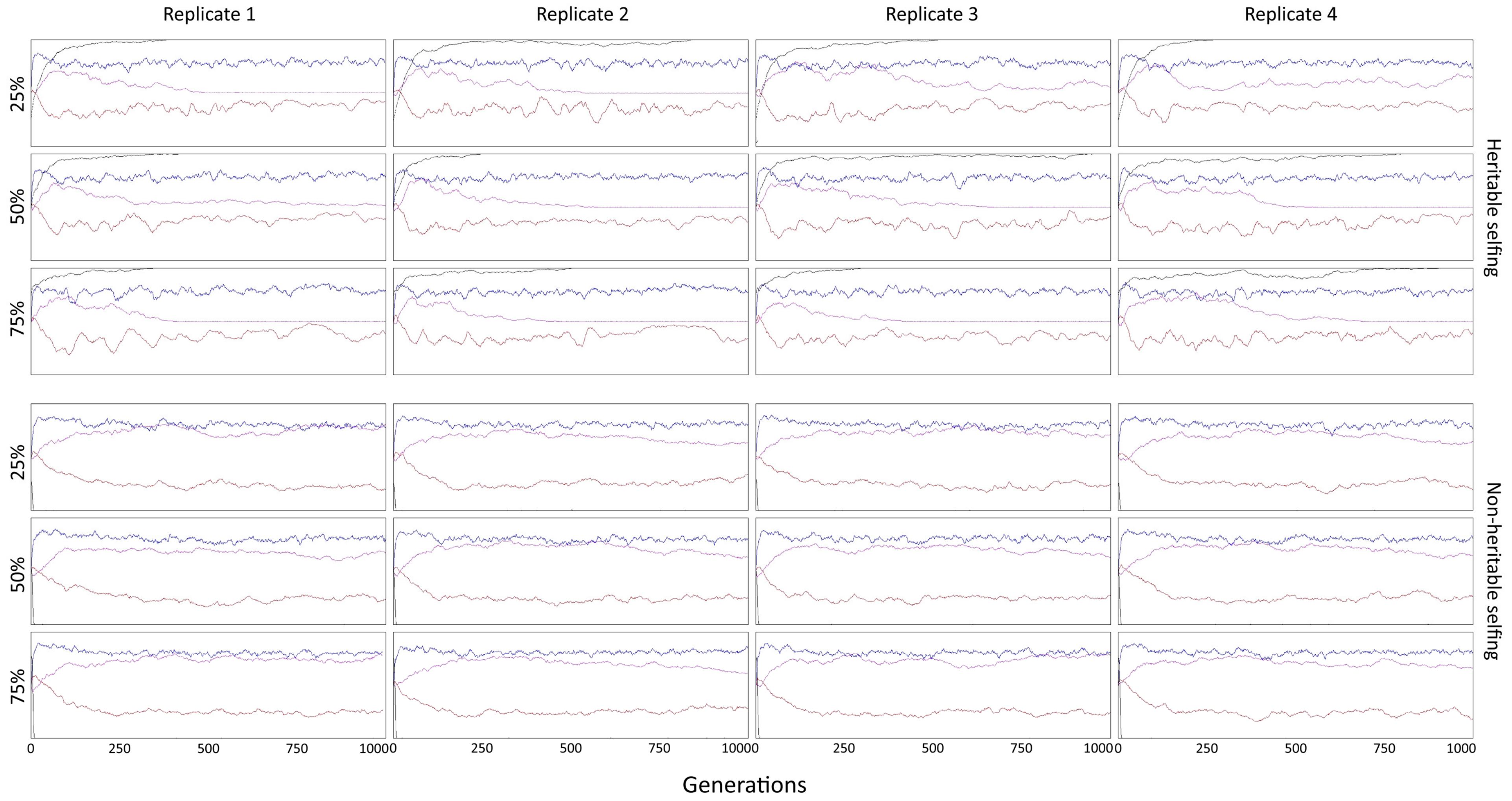

Supplementary Figure S6. Evolution of selfing for various initial ratios. Charts show the evolution of a plant's population and an herbivore's population interacting with each other. The black lines represent the selfing rate (proportion of selfers in non-heritable, and proportion of selfing alleles in heritable selfing). The blue lines show the ratio of herbivores anti-resistance (alleles), the magenta lines the ratio of tolerance (alleles), and the red lines the ratio of resistance (alleles). The proportions at left (25%, 50%, 75%) indicate the initial selfing ratio and the replicates constitute independent

simulation runs with the same parameters but different random number seeds. Notice the similitude of evolutionary histories among replicates but also among initial selfing rates.
